# Electronic Band Structure Variations in the Ceria Doped Zirconia: A First Principles Study

**DOI:** 10.3390/ma11071238

**Published:** 2018-07-19

**Authors:** Sahar Ramin Gul, Matiullah Khan, Yi Zeng, Maohua Lin, Bo Wu, Chi-Tay Tsai

**Affiliations:** 1 College of Materials Science and Engineering, and Key Laboratory of Eco-materials Advanced Technology (Fuzhou University), Fujian Province University, Fuzhou 350100, China; srgmk14@gmail.com (S.R.G.); mlin2014@fau.edu (M.L.); 2 State Key Lab of High Performance Ceramics and Superfine Microstructure, Shanghai Institute of Ceramics, Chinese Academy of Sciences, Shanghai 200050, China; 3 Department of Physics, Kohat University of Science and Technology (KUST), Kohat 26000, Pakistan; 4 Department of Ocean and Mechanical Engineering, Florida Atlantic University, Boca Raton, FL 33431, USA; tsaict@fau.edu

**Keywords:** ceria-zirconia solid solution, thermal barrier coatings, band structure, optical response, first principle

## Abstract

Using first principle calculations, the effect of Ce with different doping concentrations in the network of Zirconium dioxide (ZrO_2_) is studied. The ZrO_2_ cell volume linearly increases with the increasing Ce doping concentration. The intrinsic band gap of ZrO_2_ of 5.70 eV reduces to 4.67 eV with the 2.08% Ce doping. In 4.16% cerium doped ZrO_2_, the valence band maximum and conduction band minimum come closer to each other, about 1.1 eV, compared to ZrO_2_. The maximum band gap reduction of ZrO_2_ is observed at 6.25% Ce doping concentration, having the value of 4.38 eV. No considerable shift in the band structure is found with further increase in the doping level. The photo-response of the ZrO_2_ is modulated with Ce insertion, and two distinct modifications are observed in the absorption coefficient: an imaginary part of the dielectric function and conductivity. A 2.08% Ce-doped ZrO_2_ modeled system reduces the intensities of peaks in the optical spectra while keeping the peaks of intrinsic ZrO_2_. However, the intrinsic peaks related to ZrO_2_ completely vanish in 4.16%, 6.25%, 8.33%, and 12.5% Ce doped ZrO_2_, and a new absorption hump is created.

## 1. Introduction

Recently, zirconia (ZrO_2_) based ceramic materials got increasing attention from researchers due their wide range of applications, including thermal barrier coatings (TBC), solid oxide fuel cells, catalysis, and energy conversion [1,2,3]. In thermal barrier coatings, zirconia in its pure form faces two main problems. The first one is its phase transformation from tetragonal to monoclinic, leading to volume expansion, cracking, and failure. Secondly, its thermal conductivity is not sufficiently low, and it could be lowered by mixing it with other oxides, including CeO_2_, Sc_2_O_3_, and In_2_O_3_. Moreover, the mixing of ZrO_2_ with aforementioned oxides stabilizes ZrO_2_ at high temperature [4,5].

Ceria (CeO_2_) is widely used in the modern catalytic industry, automotive exhaust catalysis, solid oxide fuel cells, and water-gas shift (WGS) reactions [6,7]. For an oxygen storage material, it is used as a component in automotive three-way catalysts. The oxygen storage capabilities of CeO_2_ are found to degrade at high temperature, which strongly limits its applications. It is reported that the thermal stability of CeO_2_ can be improved by mixing it with zirconium oxide [8,9,10].

Charge compensation is essential in the case of substitutional doping. Ce^4+^ and Zr^4+^ both are tetravalent ions, and the substitution of Zr^4+^ by Ce^4+^ would not lead to any charge imbalance. Thus, the cerium insertion in the network of ZrO_2_ would reduce the thermal conductivity while creating minimum structure distortion. Experimentally, it is proven that Ce mixing with ZrO_2_ can lead to improved thermal stability at higher temperature, along with lowering thermal conductivity [11]. On the other hand, the addition of Zr into the bulk of CeO_2_ can improve the oxygen storage capacity of a ceria-zirconia solid solution, and it can broaden the application spectrum of CeO_2_. Zr doping in the structure of CeO_2_ is widely studied. Andersson et al. [10] used ab-initio calculations to calculate the redox thermodynamics and kinetics of CeO_2_ mixed with ZrO_2_. It was found that Zr^4+^ doping decreased the vacancy formation energy and the migration barrier in CeO_2_ [10]. Yang [6] and his co-workers studied the effect of Zr doping on the redox properties of CeO_2_. Zr doping substantially lowered the formation energy of an O vacancy when the vacancy was created next to the Zr dopant [6].

With the Ce doping concentration Ce_1−x_Zr_x_O_2_ (x = 0.25, 0.5, 0.75, 1), Tian et al. [12] reported the replacement of Ce by Zr, leading to the formation of a pseudo-cubic fluorite-type structure. Moreover, increasing the Zr doping concentration linearly decreased the lattice parameters and cell volume of CeO_2_ [12]. Because it is considered a solid solution, it would be important to increase the doping concentration of Ce in the cubic ZrO_2_ and investigate its effect on the structure and properties. Moreover, Ce doping in the structure of cubic ZrO_2_ should be studied in detail.

In this paper, Ce doping in ZrO_2_ has been studied with the Ce doping concentration of 2.08%, 4.16%, 6.25%, 8.33%, and 12.5%, corresponding to the Ce/Zr ratio of 6.25%, 12.5%, 18.75%, 25%, and 37.5%, respectively. Furthermore, the effect of Ce doping on the geometrical structure, electronic band structure, and optical properties is elucidated.

## 2. Methodology

The Kohn-Sham density functional theory (DFT) calculations were performed with Materials Studio 8.0 [13]. The valence electronic states were expanded in a basis of plane waves, and the oscillating wave function of core electrons were represented by a projected augmented wave approach. Moreover, the special Perdew-Burke-Ernzerhof functional for solids (PBEsol) [14,15] was used as an exchange correlation functional with ultrasoft pseudopotential [16]. For the plane–wave set, a cut-off energy of 400 eV and 3 × 3 × 3 k-points mesh was utilized. Simulated models were optimized with respect to cell parameters and atomic positions using the Broyden, Fletcher, Goldfarb, Shanno (BFGS) minimization scheme under the maximum force of 0.01 eV/Å. Furthermore, the stress tensor on the cell was less than 0.02 GPa [17,18].

**Figure 1:**
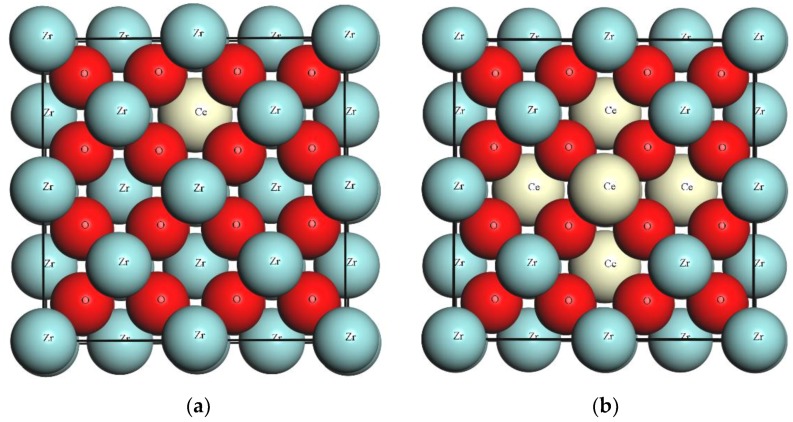
Ce incorporated zirconia structure (**a**) CeZr-1, and (**b**) CeZr-6.

A conventional 12 atoms cubic unit cell of zirconia (space group Fm-3m), extended to 2 × 2 × 1 repetition forming a 48 atom supercell, was utilized. Cerium (Ce) doped zirconia models were introduced by replacing a regular lattice Zr atom with a Ce atom. The cerium doping concentration can be increased by increasing the number of Ce atoms substituting the Zr sites. The Ce doped zirconia modeled systems and their corresponding Ce doping concentration and Ce/Zr ratio are summarized in Table 1. The CeZr-1 and CeZr-6 models are visualized in Figure 1.

## 3. Results and Discussion

### 3.1. The Effect of Ce Inclusion on the ZrO_2_ Structure

The optimized lattice parameters and cell volumes of the zirconia structure are summarized in Table 2. The pure ZrO_2_ system has a = 10.158 Å, b = 10.158 Å (double values because of 2 × 2 × 1 supercell), and c = 5.0796 Å. The optimized lattice parameters are in close agreement with the available calculated data [12,19] and experimental values [20]. Substituting Ce at Zr sites induces slight change in the lattice parameters in the form of elongation. Table 2 depicts that as the content of Ce increases, the elongation in the lattice parameters increases. The higher ionic radii of Ce^4+^ = 0.97 Å in comparison to Zr^4+^ = 0.84 Å is responsible for this change [6]. Furthermore, the cell volume of zirconia gradually increased with the increasing Ce concentration.

**Figure 2:**
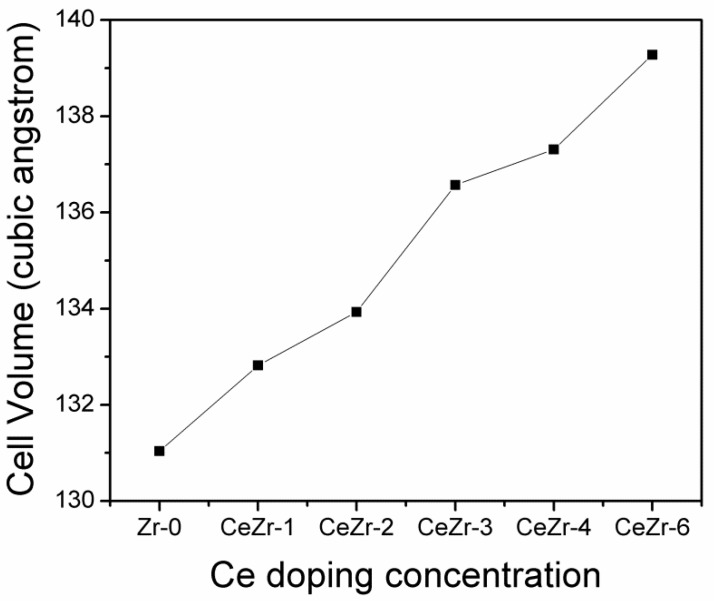
Variations in the cell volume with increasing Ce doping concentration.

The behavior of the cell volume with the Ce doping concentration is displayed in Figure 2. A linear increase in the unit cell volume is found with the increasing Ce doping concentration. Cerium (0.97 Å) insertion at Zr (0.84 Å) sites is responsible for the increase in cell volume.

The average bond lengths of the optimized Ce doped ZrO_2_ modeled systems are summarized in Table 3. The zirconia system is optimized with the O-Zr and O-O bond lengths of 2.1993 Å and 2.5395 Å, respectively. The calculated data is in good agreement with the theoretical [12] and experimental [21] findings. Substituting Ce at Zr sites elongates the O-Zr and O-O bond lengths. Table 3 depicts that the O-Zr and O-O bond lengths linearly increase with an increasing Ce doping concentration. The increasing trend of the O-Zr and O-O bond lengths is ascribed to the difference in the ionic radii of Ce^4+^ and Zr^4+^. It is interesting to note that the O-Ce bond length initially showed an increasing trend, which became nearly constant for CeZr-2, CeZr-3, CeZr-4, and CeZr-6.

### 3.2. The Effect of Ce Inclusion on the Electronic Band Structure of Zirconia

**Figure 3:**
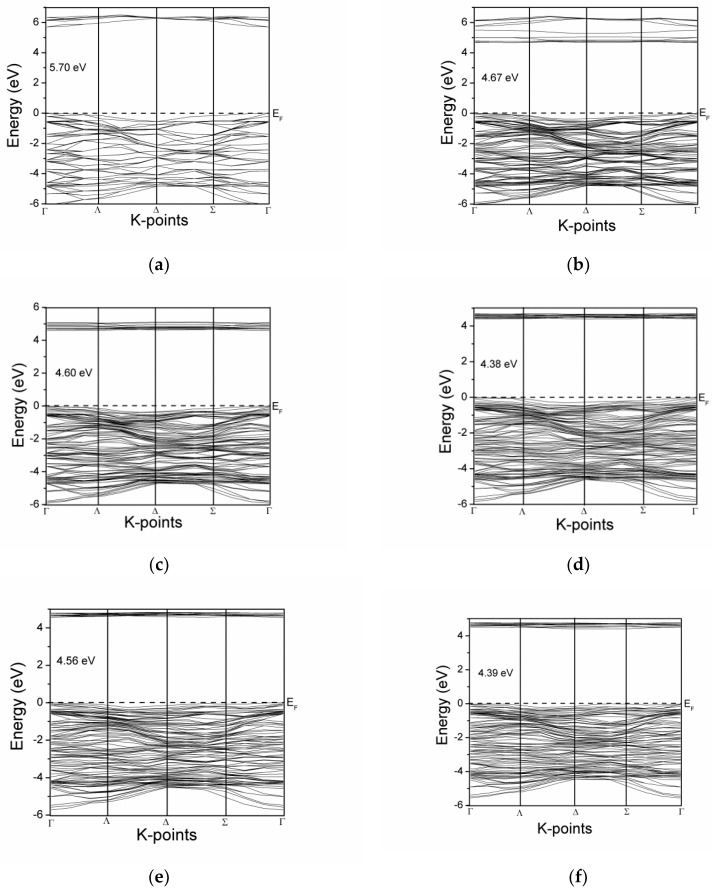
Band structure comparison of simulated models: (**a**) Zr-0, (**b**) CeZr-1, (**c**) CeZr-2, (**d**) CeZr-3, (**e**) CeZr-4, and (**f**) CeZr-6.

The band structures of Ce doped ZrO_2_ modeled systems are shown in Figure 3. Generalized gradient approximation (GGA) calculations underestimate the band gap due to the well-known shortcoming of density functional theory (DFT) [22]. For cubic ZrO_2_, the calculated band gap is 3.307 eV, which is underestimated compared to the experimental value of 5.70 eV. A constant scissor operator is applied for all investigated structures for making the band structure comparable to the experimental value [23]. Intrinsic insulating behavior is clarified from Figure 3a, displaying the Fermi level at the top of the valence band. No isolated states appear in the forbidden region due to Ce doping. Cerium inclusion in the ZrO_2_ network modified the band structure of ZrO_2_. No specific trend is observed in the band gap of ZrO_2_ with an increasing Ce doping concentration. The band gap of CeZr-1 is reduced to 4.67 eV, compared to the 5.70 eV for Zr-0. The valence band and conduction band come closer to each other, giving a gap of 4.60 eV in the case of CeZr-2. A minimum band gap has been observed for the CeZr-3 model, although CeZr-4 and CeZr-6 possess higher Ce doping concentrations compared to CeZr-3. With the Fermi level at valence band maximum, the distance between the top of the valence and bottom of the conduction band at the gamma point in CeZr-4 and CeZr-6 systems are 4.56 eV and 4.39 eV, respectively.

**Figure 4:**
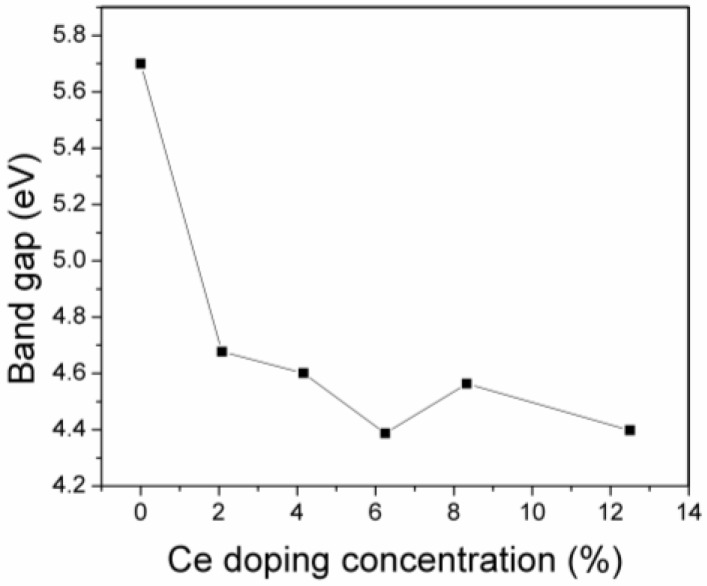
Variations in the band gap of Ce- ZrO_2_ system with increasing Ce doping concentration.

Ce doping modified the intrinsic band structure of ZrO_2_, and the modifications in the band gap of Ce doped ZrO_2_ modeled systems are depicted in Figure 4. One should note that Ce doping reduced the band gap of ZrO_2_; however, the different doping systems have different reduction values. The 6.25% Ce doped ZrO_2_ results in a maximum reduction of 1.32 eV in the ZrO_2_ band gap. No linear modification in the band gap is found with the increasing Ce doping concentration. The maximum Ce doping concentration reported in this paper of 12.5% reduced the band gap around 1.31 eV, compared to pure zirconia.

**Figure 5:**
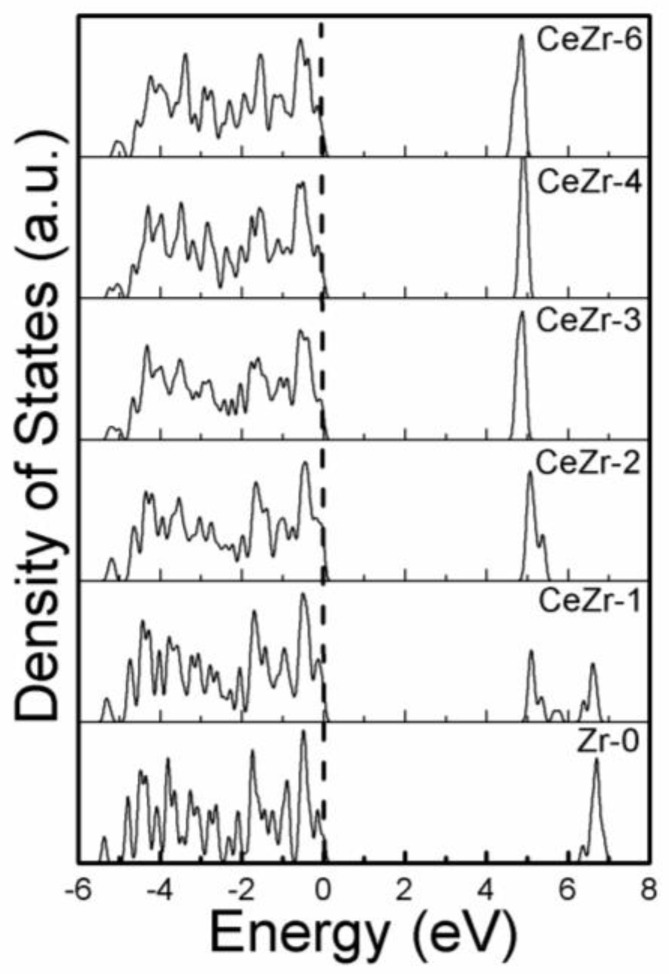
Density of states of simulated models and their comparison. The vertical dashed line represents the Fermi level.

The density of states is calculated for elaborating the electronic structure of the modeled systems in detail. As verified from Figure 5, the recent calculations show no gap states between the valence and conduction band. The only modification that Ce doping performed is the shifting of the valence band edge and conduction band closer to each other. The band gap is modified due to Ce insertion in the ZrO_2_ network, and all Ce doped ZrO_2_ modeled systems depict a reduced band gap. The density of states findings are consistent with the reported data [12].

### 3.3. The Effect of Ce Inclusion on the Photo-Response of Zirconia

The photo-response of zirconia originates from the interactions of photons with the electrons in ZrO_2_, leading to the transition of electrons between the occupied and unoccupied states. CASTEP calculates the real and imaginary part of the dielectric function, which are further used to calculate the other optical properties like absorption, reflectivity, and conductivity [24,25]. The imaginary part of the dielectric function given in Equation (1) is used to calculate the optical absorption (Equation (2)).

(1) $$\varepsilon_{2}(\hbar \omega )=\frac{2\pi e^{2}}{\Omega \varepsilon_{o}}{\sum_{c,v}\sum_{k}\left|\langle \psi_{k}^{c}|\hat{u}.r|\psi_{k}^{v}\rangle \right|}^{2}\delta (E_{k}^{c}-E_{k}^{v}-\hbar \omega )\;$$

(2) $$\alpha (\omega )=\sqrt{2}\omega {\left[\sqrt{\varepsilon_{1}^{2}(\omega )+\varepsilon_{2}^{2}(\omega )}\varepsilon_{1}(\omega )\right]}^{\frac{1}{2}}\;$$

The Ω, *v*, *c*, *ω*, and *k* in Equation (1) represent the volume of the elementary cell, valence band, conduction band, frequency, and the vector defining the polarization direction of the electric field. Absorption coefficient (Equation (2)) is represented by α(ω).

**Figure 6:**
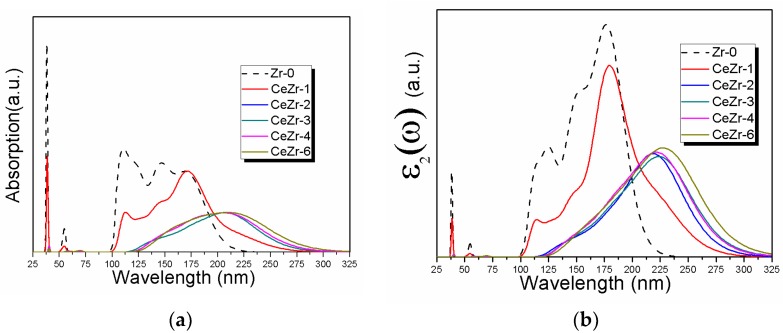

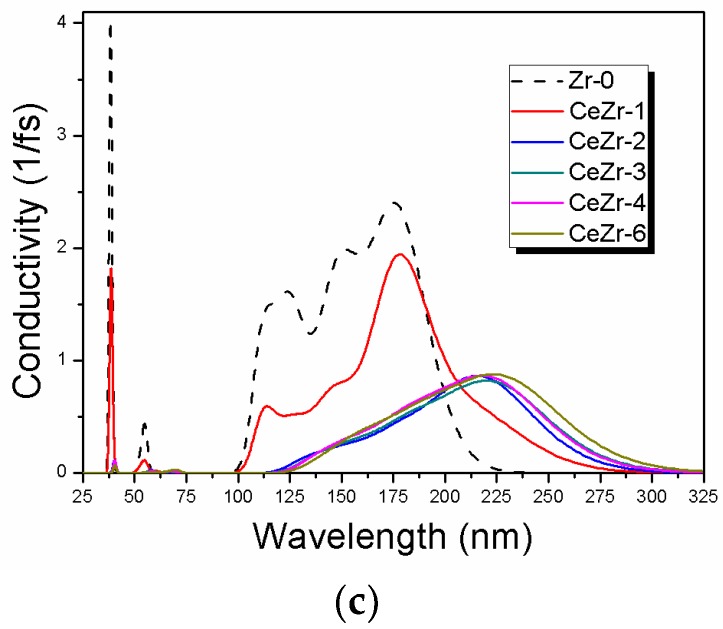
Response of the simulated models to upcoming photons with different wavelengths: (**a**) absorption, (**b**) imaginary part of dielectric function, and (**c**) conductivity.

The optical properties of Ce doped ZrO_2_ modeled systems are displayed in Figure 6. Due to the intrinsic wide band gap (5.70 eV for ZrO_2_), pure ZrO_2_ is found to absorb high energy photons. As clarified from Figure 6a, pure zirconia has a strong absorption peak, around 38.6 nm and 54.9 nm. Moreover, a wide absorption band is observed starting from 101 nm and ending at 224.1 nm. These peaks might originate from the transitions of electrons between O 2p and Zr 3d states. Ce doping modified the optical response of ZrO_2_. Increasing Ce doping concentration created substantial change in the absorption curves of ZrO_2_. The CeZr-1 depicted an absorption peak at 39.4 and 54.0 nm, with less intensity compared to pure zirconia. The broad absorption band in case of CeZr-1 is shifted to longer wavelengths ending at 274.8 nm. The changes induced in the optical absorption spectra of CeZr-1 are attributed to the insertion of Ce 4f states in the band structure. It is interesting to note that the models CeZr-2, CeZr-3, CeZr-4, and CeZr-6 exhibit different behavior to upcoming photons. The peaks around 39 nm and 54 nm completely disappeared. Moreover, the broad peak is shifted to longer wavelengths starting at 122.7 nm and ending at 290.2 nm. One should note from Figure 6a that there is very small difference between the absorption spectra of CeZr-2, CeZr-3, CeZr-4, and CeZr-6. The shifting of the absorption peaks and the creation/disappearance of new/existing peaks are ascribed to the states introduced due to Ce doping. The imaginary of dielectric function is shown in Figure 6b. Peaks for Zr-0 and relatively smaller peaks for CeZr-1 are found at 38.6 nm, 54.0 nm, and 176.1 nm attributed to the excitations of electrons from O 2p to Zr 3d states. The optical absorption drastically changed in CeZr-2, CeZr-3, CeZr-4, and CeZr-6 modeled systems. The peaks around 38.6 nm and 54 nm disappeared, and the absorption hump is found centering at around 225 nm. Electronic transitions are responsible for the optical spectra. One should note that the band structure of the doped systems changed due to the creation of Ce 4f states, leading to modification in the optical spectra. The peak hump around 225 nm is attributed to excitations of electrons between O 2p to Ce 4f and Zr 3d states. The absorption spectra and the imaginary part of the dielectric function verify the band structure findings. Figure 6c summarizes the conductivity of the simulated models. Computational results of the conductivity of Zr-0, as seen from Figure 6c, show peaks at 38.6 nm and 54 nm. Following the peaks, a broad absorption is found starting at 99.2 nm and ending at 227.7 nm. The 2.08% Ce doped ZrO_2_ displays peaks with less intensity compared to Zr-0 at 38.6 nm and 54 nm. Moreover, the broad absorption hump is also found with reduced intensity. It is interesting to note that the hump starting at 101 nm for CeZr-1 is shifted to higher wavelengths ending at 275.7 nm. CeZr-2, CeZr-3, CeZr-4, and CeZr-6 demonstrate a single absorption band starting at 124.5 nm and ending at around 300 nm.

## 4. Conclusions

Ce doping significantly modified the electronic band structure and photo-response of ZrO_2_. The cell volume of the basic unit cell of ZrO_2_ exhibited linear variations with the Ce doping concentration. The electronic band gap of ZrO_2_ was reduced considerably by doping ZrO_2_ with Ce. A maximum reduction of about 1.32 eV was found for the CeZr-3 model, with a Ce doping concentration of 6.25%. The optical properties investigation revealed that the intrinsic peaks of ZrO_2_ were maintained at low doping levels, and these peaks completely vanished at high doping levels, leading to the creation of a new absorption band. The Ce inclusion in ZrO_2_ is expected to reduce the thermal conductivity, which would widen the utilization of Ce doped ZrO_2_ in thermal barrier coating applications.

## Figures and Tables

**Table 1:** The Ce doping concentration and Ce/Zr ratio of simulated models.

S. No.	Model	Representation	Ce Doping Concentration (%)	Ce/Zr Ratio (%)
1	Zr_16_O_32_	Zr-0	0	100
2	CeZr_15_O_32_	CeZr-1	2.08	6.25
3	Ce_2_Zr_14_O_32_	CeZr-2	4.16	12.5
4	Ce_3_Zr_13_O_32_	CeZr-3	6.25	18.75
5	Ce_4_Zr_12_O_32_	CeZr-4	8.33	25.0
6	Ce_6_Zr_10_O_32_	CeZr-6	12.5	37.5

**Table 2:** Lattice parameters and unit cell volumes of Ce doped zirconia modeled systems.

Model	a (Å)	b (Å)	c (Å)	Cell Volume (Å^3^)
Zr-0	5.0790	5.0790	5.0796	131.0392
Zr-0 (calculations [12])	5.0654	5.0654	5.0654	129.974
Zr-0 (experiments [20])	-	-	5.090	-
CeZr-1	5.0959	5.0945	5.0925	132.8176
CeZr-2	5.1179	5.1179	5.1132	133.9314
CeZr-3	5.1489	5.1520	5.1482	136.5704
CeZr-4	5.1582	5.1582	5.1604	137.3086
CeZr-6	5.1886	5.1886	5.1733	139.2799

**Table 3:** Bond lengths (averaged) of the simulated systems.

S. No.	Model	O-Zr (Å)	O-O (Å)	O-Ce (Å)
1	Zr-0	2.1993	2.5395	-
2	CeZr-1	2.2004	2.5430	2.2885
3	CeZr-2	2.2049	2.5507	2.2919
4	CeZr-3	2.2127	2.5643	2.2992
5	CeZr-4	2.2144	2.5650	2.2944
6	CeZr-6	2.2199	2.5813	2.3006

## References

[1] Albert M.I., Yingna D., Yoshitaka U. (2017). Effect of cation dopants in zirconia on interfacial properties in nickel/zirconia systems: An atomistic modeling study. J. Phys. Condens. Matter.

[2] Wang Y., Xu F., Gauvin R., Kong M., Khan M., Liu Z., Zeng Y. (2017). Growth modes for monoclinic yttria-stabilized zirconia during the martensitic transformation. J. Am. Ceram. Soc..

[3] Vasilopoulou M., Georgiadou D.G., Soultati A., Boukos N., Gardelis S., Palilis L.C., Fakis M., Skoulatakis G., Kennou S., Botzakaki M. (2014). Atomic-Layer-Deposited Aluminum and Zirconium Oxides for Surface Passivation of TiO_2_ in High-Efficiency Organic Photovoltaics. Adv. Energy Mater..

[4] Padture N.P., Gell M., Jordan E.H. (2002). Thermal Barrier Coatings for Gas-Turbine Engine Applications. Science.

[5] Cao X.Q., Vassen R., Stoever D. (2004). Ceramic materials for thermal barrier coatings. J. Eur. Ceram. Soc..

[6] Yang Z., Woo T.K., Hermansson K. (2006). Effects of Zr doping on stoichiometric and reduced ceria: A first-principles study. J. Chem. Phys..

[7] Chen H.-T., Chang J.-G. (2010). Oxygen vacancy formation and migration in Ce_1−x_Zr_x_O_2_ catalyst: A DFT+U calculation. J. Chem. Phys..

[8] Murota T., Hasegawa T., Aozasa S., Matsui H., Motoyama M. (1993). Production method of cerium oxide with high storage capacity of oxygen and its mechanism. J. Alloys Compd..

[9] Fornasiero P., Dimonte R., Rao G.R., Kaspar J., Meriani S., Trovarelli A., Graziani M. (1995). Rh-Loaded CeO_2_-ZrO_2_ Solid-Solutions as Highly Efficient Oxygen Exchangers: Dependence of the Reduction Behavior and the Oxygen Storage Capacity on the Structural-Properties. J. Catal..

[10] Andersson D.A., Simak S.I., Skorodumova N.V., Abrikosov I.A., Johansson B. (2007). Redox properties of CeO_2_–MO_2_ (M=Ti, Zr, Hf, or Th) solid solutions from first principles calculations. Appl. Phys. Lett..

[11] Yang F., Zhao X., Xiao P. (2012). The effects of temperature and composition on the thermal conductivities of [(ZrO_2_)_1−x_(CeO_2_)_x_]_0.92_(Y_2_O_3_)_0.08_ (0 ≤ x ≤ 1) solid solutions. Acta Mater..

[12] Tian D., Zeng C., Wang H., Luo H., Cheng X., Xiang C., Wei Y., Li K., Zhu X. (2016). Performance of cubic ZrO_2_ doped CeO_2_: First-principles investigation on elastic, electronic and optical properties of Ce_1−x_Zr_x_O_2_. J. Alloys Compd..

[13] Khan M., Cao W., Chen N., Iqbal M.Z. (2013). Ab-initio calculations of synergistic chromium–nitrogen codoping effects on the electronic and optical properties of anatase TiO_2_. Vacuum.

[14] Skorodumova N.V., Baudin M., Hermansson K. (2004). Surface properties of CeO_2_ from first principles. Phys. Rev. B.

[15] Perdew J.P., Ruzsinszky A., Csonka G.I., Vydrov O.A., Scuseria G.E., Constantin L.A., Zhou X., Burke K. (2008). Restoring the Density-Gradient Expansion for Exchange in Solids and Surfaces. Phys. Rev. Lett..

[16] Khan M., Cao W., Ullah M. (2013). Ab initiocalculations for the electronic and optical properties of Y-doped anatase TiO_2_. Phys. Status Solidi.

[17] Pfrommer B.G., Côté M., Louie S.G., Cohen M.L. (1997). Relaxation of Crystals with the Quasi-Newton Method. J. Comput. Phys..

[18] Ramin G.S., Matiullah K., Zeng Y., Bo W. (2017). Structural, electronic and optical properties of non-compensated and compensated models of yttrium stabilized zirconia. Mater. Res. Express.

[19] Garcia J.C., Scolfaro L.M.R., Lino A.T., Freire V.N., Farias G.A., Silva C.C., Alves H.L., Rodrigues S.C.P., da Silva E.F. (2006). Structural, electronic, and optical properties of ZrO_2_ from ab initio calculations. J. Appl. Phys..

[20] Stefanovich E.V., Shluger A.L., Catlow C.R.A. (1994). Theoretical study of the stabilization of cubic-phase ZrO_2_ by impurities. Phys. Rev. B.

[21] French R.H., Glass S.J., Ohuchi F.S., Xu Y.N., Ching W.Y. (1994). Experimental and theoretical determination of the electronic structure and optical properties of three phases of ZrO_2_. Phys. Rev. B.

[22] Khan M., Cao W. (2013). Cationic (V, Y)-codoped TiO_2_ with enhanced visible light induced photocatalytic activity: A combined experimental and theoretical study. J. Appl. Phys..

[23] Khan M., Xu J., Chen N., Cao W. (2012). First principle calculations of the electronic and optical properties of pure and (Mo, N) co-doped anatase TiO_2_. J. Alloys Compd..

[24] Sun J., Wang H.-T., He J., Tian Y. (2005). Ab initio investigations of optical properties of the high-pressure phases of ZnO. Phys. Rev. B.

[25] Khan M., Xu J., Chen N., Cao W. (2012). Electronic and optical properties of pure and Mo doped anatase TiO_2_ using GGA and GGA+U calculations. Phys. B Condens. Matter.

